# Charge-tagging liquid chromatography–mass spectrometry methodology targeting oxysterol diastereoisomers

**DOI:** 10.1016/j.chemphyslip.2017.04.004

**Published:** 2017-10

**Authors:** William J. Griffiths, Thomas Hearn, Peter J. Crick, Jonas Abdel-Khalik, Alison Dickson, Eylan Yutuc, Yuqin Wang

**Affiliations:** Swansea University Medical School, ILS1 Building, Singleton Park, Swansea, SA2 8PP, UK

**Keywords:** ACOX2, acyl-coenzyme A oxidase 2, AMACR, alphamethylacyl-CoA racemase, CTX, cerebrotendinous xanthomatosis, CYP, cytochrome P450, EADSA, enzyme-assisted derivatisation for steroid analysis, ESI, electrospray ionisation, GC, gas chromatography, GP, Girard P reagent, HSD, hydroxysteroid dehydrogenase, LC, liquid chromatography, LIT, linear ion trap, MRM, multiple reaction monitoring, MS, mass spectrometry, MS^n^, MS with multistage fragmentation, NPC, Niemann Pick type C, RIC, reconstructed ion chromatogram, SLOS, Smith-Lemli-Opitz syndrome, SRM, standard reference material, 3β,7α-diHCA(25R), 3β,7α-dihydroxycholest-5-en-(25R)26-oic acid, 3β,7α-diHCA(25S), 3β,7α-dihydroxycholest-5-en-(25S)26-oic acid, 3β,7α-diH-Δ^5^-BA, 3β,7α-dihydroxychol-5-enoic acid, 3β,7β-diHCA(25R), 3β,7β-dihydroxycholest-5-en-(25R)26-oic acid, 3β,7β-diHCA(25S), 3β,7β-dihydroxycholest-5-en-(25S)26-oic acid, 3β,7β-diH-Δ ^5^-BA, 3β,7β-dihydroxychol-5-enoic acid, 7-OC, 7-oxocholesterol, 7α-HC, 7α-hydroxycholesterol, 7α-HCO, 7α-hydroxycholest-4-en-3-one, 7αH,3O-CA(25R), 7α-hydroxy-3-oxocholest-4-en-(25R)26-oic acid, 7αH,3O-CA(25S), 7α-hydroxy-3-oxocholest-4-en-(25S)26-oic acid, 7αH,3O-Δ^4^-BA, 7α-hydroxy-3-oxochol-4-enoic acid, 7β-HC, 7β-hydroxycholesterol, 24R-HC, 24R-hydroxycholesterol, 24S-HC, 24S-hydroxycholesterol, (25R)26-HC, (25R)26-hydroxycholesterol, 25-HC, 25-hydroxycholesterol, Sterol, Hydroxycholesterol, Cholestenoic acid, Epimer, Derivatisation

## Abstract

The introduction of a hydroxy group to the cholesterol skeleton introduces not only the possibility for positional isomers but also diastereoisomers, where two or more isomers have different configurations at one or more of the stereocentres but are not mirror images. The differentiation of diastereoisomers is important as differing isomers can have differing biochemical properties and are formed via different biochemical pathways. Separation of diasterioisomers is not always easy by chromatographic methods Here we demonstrate, by application of charge-tagging and derivatisation with the Girard P reagent, the separation and detection of biologically relevant diastereoisomers using liquid chromatography − mass spectrometry with multistage fragmentation.

## Highlights

• EADSA and LC–MS allows the differentiation of sterol diastereoisomers.
• 24S- and 24R-hydroxycholesterol are present in human plasma.
• Four diastereoisomers of 3β,7-dihydroxycholest-5-enoic acid are found in human plasma.
• 3β,7α- and 3β,7β-dihydroxychol-5-enoic acids are found in human plasma.
• 7α- and 7β- epimers give distinguishable MS^3^ spectra.

## Graphical abstract

**Figure:**
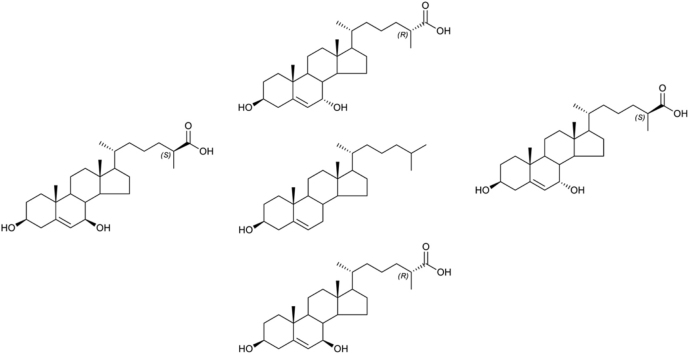

## 1 Introduction

**Fig. 1:**
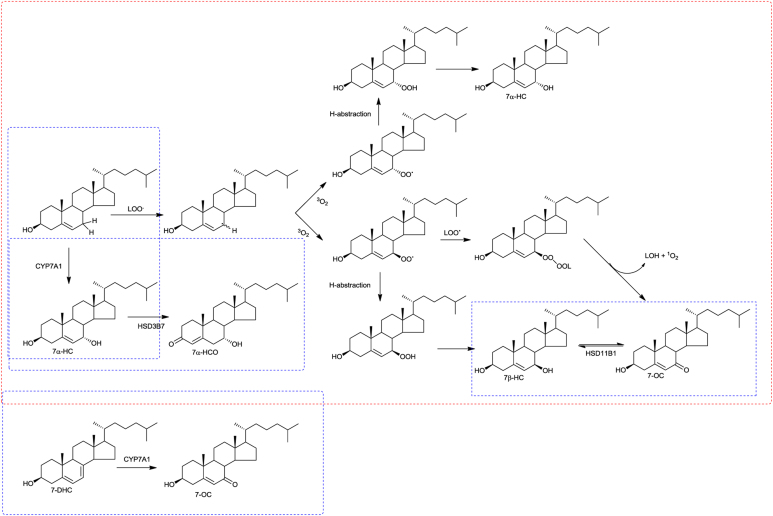
Formation of 7-hydroxycholesterol (7-HC) and 7-oxocholesterol (7-OC) by radical initiated and enzymatic reactions. LOO^.^ corresponds to a lipid hydroperoxy radical and LOH to a hydroxy lipid. Enzyme catalysed reactions are shown in the blue boxes and radical initiated pathways in the red box. (For interpretation of the references to colour in this figure legend, the reader is referred to the web version of this article.)

The most commonly observed diastereoisomers derived from cholesterol are 7α-hydroxycholesterol and 7β-hydroxycholesterol (7α-HC and 7β-HC). These can be formed via free radical oxidation by initial abstraction of the C-7 hydrogen followed by reaction with oxygen to form a peroxyradical, hydrogen abstraction to form a peroxide and ultimately reduction to 7α- or 7β-HC (Murphy and Johnson, 2008) (Fig. 1). These reactions may proceed in air during sample handling or in the biological systems, e.g. in the lysosomal storage disease Niemann Pick type C (NPC) (Alvelius et al., 2001). 7α-HC is also formed enzymatically from cholesterol by the enzyme cytochrome p450 7A1 (CYP7A1) in the first step of the neutral pathway of bile acid biosynthesis (Russell, 2003), while 7β-HC can be formed from 7-oxocholesterol (7-OC) in a reaction catalysed by hydroxysteroid dehydrogenase 11B1 (HSD11B1) (Hult et al., 2004, Larsson et al., 2007, Mitic et al., 2013). 7-OC can be formed from the cholesterol precursor 7-dehydrocholesterol (7-DHC) in a reaction also catalysed by CYP7A1 (Shinkyo et al., 2011) and also by a free radical oxidation mechanism from cholesterol (Fig. 1). Both 7-OC and 7β-HC are abundant in the disease Smith Lemli Opitz Syndrome (SLOS) where 7-DHC, the CYP7A1 substrate, is present at elevated concentrations (Griffiths et al., 2016). To understand the origin of 7α-HC and 7β-HC it is necessary to differentiate between them. While the epimers 7α-HC and 7β-HC can be resolved by gas chromatography (GC) (Dzeletovic et al., 1995) this is not always the case with liquid chromatography (LC) (McDonald et al., 2012).

Less commonly considered diastereoisomers are 24S- and 24R-hydroxycholesterols (24S-HC and 24R-HC). 24S-HC is formed from cholesterol by the enzyme CYP46A1, predominantly expressed in neurons in brain, however, the origin of 24R-HC is less well established (Russell, 2003). Most GC-mass spectrometry (MS) methods are not optimised to differentiate between these epimers and it is generally assumed that all 24-HC detected is the 24S-epimer. This may not necessarily be the case.

A further stereocentre of biological importance is at C-25. During bile acid biosynthesis side-chain shortening in the peroxisome the stereochemistry at C-25 of the C_27_ acid is changed from R to S by the enzyme alphamethylacyl-CoA racemase (AMACR). This is necessary for the next step in the pathway, catalysed by the peroxisomal enzyme acyl-coenzyme A oxidase 2 (ACOX2), which introduces a double bond between C-24 and C-25, to proceed and ultimately lead to a C_24_ acid (Russell, 2003). When found in plasma, cholestenoic acids are usually assumed to have R stereochemistry at C-25.

Here we describe how we have optimised the charge-tagging technique christened enzyme-assisted derivatisation for steroid analysis (EADSA) (Griffiths et al., 2013b) to separate and identify oxysterol, cholestenoic and cholenoic acid diastereoisomers.

## 2 Materials and methods

### 2.1 Materials

Human serum and plasma samples were from waste material from previous studies in Swansea (Abdel-Khalik et al., 2017) or supplied by the Reference Institute for Bioanalytics, Bonn, Germany as a reference material for interlaboratory comparison of oxysterol measurements (Lütjohann et al., 2014). Written informed consent was obtained from all subjects in accordance with the Declaration of Helsinki. The study was conducted with institutional review board approval. The NIST standard reference material (SRM1950) was by prepared by NIST from plasma samples from 100 individuals between 40 and 50 years of age, whose ethnicity was representative of the US population and that included an equal number of men and women (Gaithersburg, MD) (Quehenberger et al., 2010).

All reagents were from sources documented in (Crick et al., 2015a, Crick et al., 2015b). Oxysterol, cholestenoic and cholenoic acid authentic standard were from Avanti Polar Lipids Inc (Alabaster, AL) or from kind donations by Professor Jan Sjövall, Karolinska Institute, Stockholm.

### 2.2 Methods

#### 2.2.1 EADSA

To enhance sensitivity, solubility in solvents used in reversed-phase chromatography and to enrich structure-informative fragmentation we employed EADSA. In brief, after separation of oxysterols and C_24_ and C_27_ acids from cholesterol and other hydrophobic sterols, an aliquot (A) of the oxysterol fraction containing the acids was oxidised with cholesterol oxidase from Streptomyces sp (Sigma-Aldrich, Dorset, UK) to convert 3β-hydroxy-5-ene structures to 3-oxo-4-enes. The oxo groups were then reacted with the Girard P reagent (GP) to generate GP-hydrazones which were separated from excess reagents on an OASIS HLB reversed phase solid phase extraction column (Waters, Elstree, UK). As some oxysterols naturally contain a 3-oxo-4-ene group e.g. 7α-hydroxycholest-4-en-3-one (7α-HCO), to avoid confusion with those oxidised by cholesterol oxidase to contain this function a separate aliquot of sample (B) was prepared in an identical fashion but in the absence of cholesterol oxidase. To allow the simultaneous analysis of aliquots (A) and (B), [^2^H_5_]GP was used to derivatise aliquots (A) and [^2^H_0_]GP to derivatise aliquots (B). The two aliquots were combined immediately before LC–MS analysis. Oxysterols derivatised with [^2^H_5_]GP will have a mass 5.0314 Da heavier than those derivatised with [^2^H_0_]GP. Note an alkaline hydrolysis step was not performed so free were oxysterols analysed not total oxysterols.

#### 2.2.2 LC–MS^n^

Derivatised oxysterols and C_24_ and C_27_ acids were separated on a Hypersil Gold reversed phase column (Thermo-Fisher Scientific, Hemel Hempstead, UK) as described in (Crick et al., 2015b) and analysed by electrospray ionisation (ESI) − MS and −MS with multistage fragmentation (MS^n^) on an Orbitrap Elite hydride linear ion trap (LIT) − Orbitrap mass spectrometer (Thermo-Fisher Scientific, Hemel Hempstead, UK). While the Orbitrap performed *m*/*z* scans at high resolution (120,000 full-width at half height definition) the LIT simultaneously performed MS^3^ scans on preselected precursor and product-ions.

## 3 Results

### 3.1 LC separation and MS^n^ fragmentation

As discussed previously (Crick et al., 2015b), GP-derivatised oxysterols fragment in the first MS/MS event (MS^2^) with the loss of the pyridine ring to give the [M-Py]^+^ fragment, [M-84]^+^ for [^2^H_5_]GP and [M-79]^+^ for [^2^H_0_]GP. [M-Py]^+^ ions then fragment in an MS^3^ (MS/MS/MS) event to give structurally informative fragment-ions showing cleavage in the ring-system, side-chain and with loss of H_2_O and CO and combinations thereof.

#### 3.1.1 7α-HC, 7β-HC and 7α-HCO

**Fig. 2:**
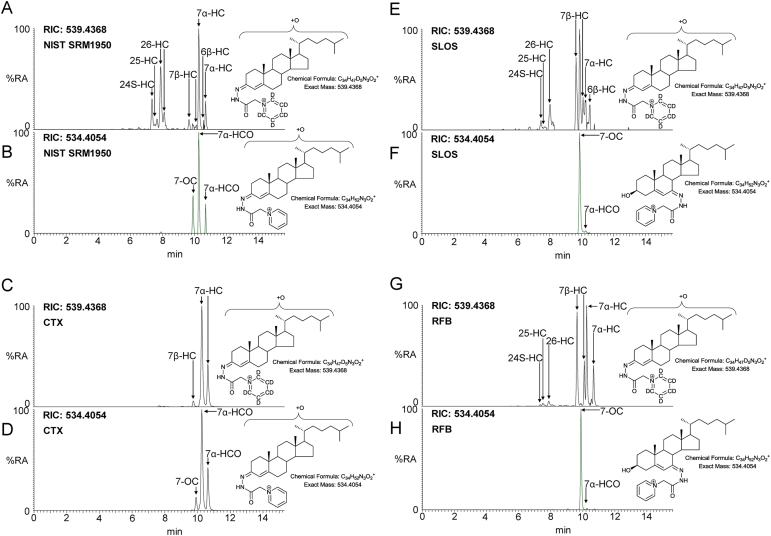
Reconstructed-ion chromatograms (RICs) displaying how 7α-HC, 7β-HC, 7α-HCO and 7-OC vary in plasma in health and disease and according to sample storage. (A) RIC from NIST SRM1950 plasma treated with cholesterol oxidase and (B) in the absence of cholesterol oxidase. (C) RIC from a typical CTX plasma sample treated with cholesterol oxidase and (D) in the absence of cholesterol oxidase. (E) RIC from a typical SLOS plasma sample treated with cholesterol oxidase and (F) in the absence of cholesterol oxidase. (G) RIC from a dried plasma sample, transported to Swansea from Germany without precaution being taken against oxidation in air, treated with cholesterol oxidase and (H) in the absence of cholesterol oxidase. Aliquots of sample prepared in the presence of cholesterol oxidase were derivatised with [^2^H_5_]GP and those prepared in the absence of cholesterol oxidase with [^2^H_0_]GP. Analyte concentrations are given in supplementary Table S1 (Crick et al., 2015b, Griffiths et al., 2016, Griffiths et al., 2013a, Theofilopoulos et al., 2014).

7α-HC and 7β-HC are chromatographically resolved in our system (Fig. 2A). The introduction of a polar hydroxyl group to the non-polar β-face of the sterol ring system reduces its hydrophobicity and retention on a C_18_ reversed-phase column. A reconstructed ion chromatogram (RIC, ± 5 ppm) from a typical serum sample is shown in Fig. 2A. Both 7β-HC and 7α-HC give double peaks as a consequence of *syn* and *anti* conformers of the GP-derivative. The RIC in Fig. 2A is for the aliquot treated with cholesterol oxidase and derivatised with [^2^H_5_]GP. Fig. 2B shows the chromatogram generated from aliquot B in the absence of cholesterol oxidase and derivatised with [^2^H_0_]GP. This chromatogram reveals compounds with a *natural* oxo group, i.e. 7α-HCO and 7-OC. While 7α-HCO can be formed *in vivo* from 7α-HC by HSD3B7 (Fig. 1), 7β-HCO is not formed from 7β-HC by this enzyme (Furster et al., 1996) and is thus absent. This, beside chromatographic resolution and differing MS^3^ spectra (see below), provides further evidence for the identity of the peaks assigned to the 7β-hydroxy epimer in chromatogram 2A. Absolute confirmation of identity was made by comparison with authentic standards. As peaks in aliquot (A) correspond to the sum of both 3β-hydroxy-5-ene and 3-oxo-4-ene compounds, while those in the (B) aliquot just to oxo compounds, quantification of 3β-hydroxy-5-ene compounds is easily performed by subtracting concentrations determined for a peak in (B) from those determined for the equivalent peak in (A). Concentration determined for peaks in (B) are those for the *natural* oxo compounds.

**Fig. 3:**
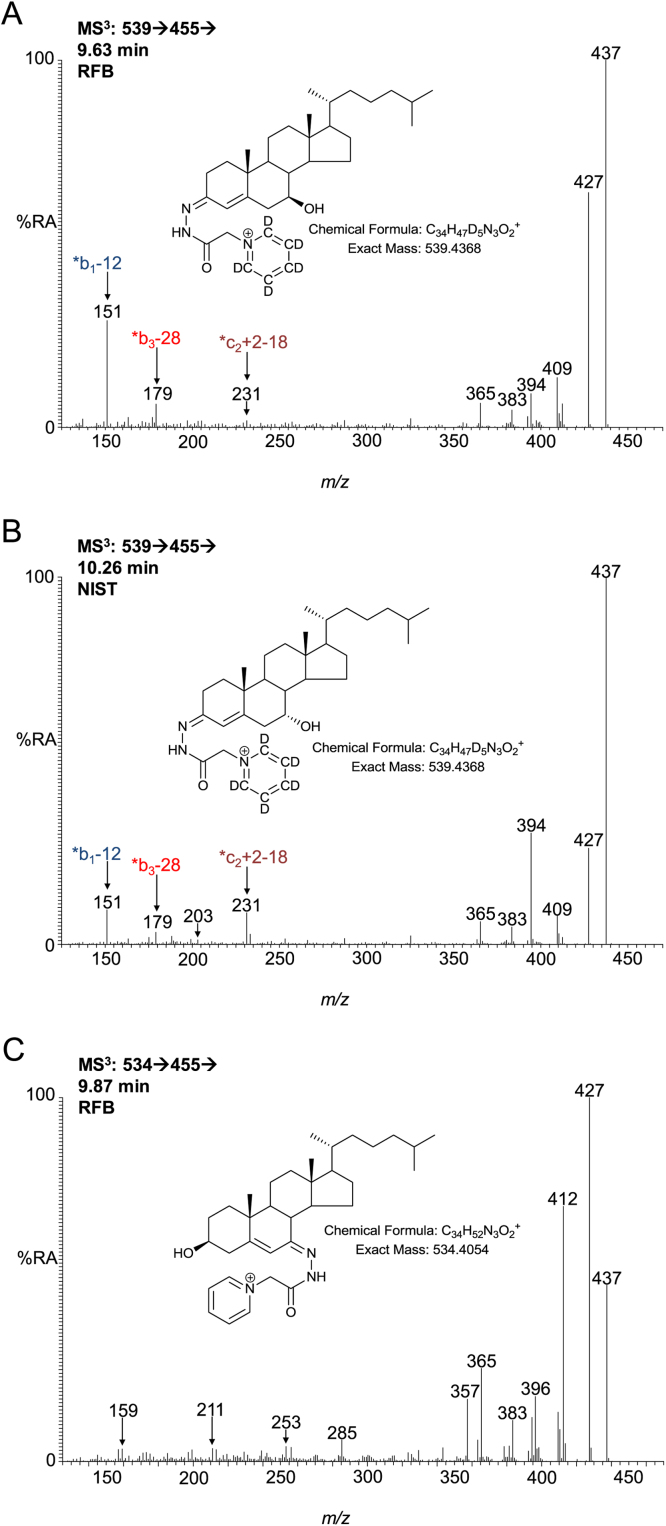
MS^3^ ([M^+^ → [M-Py]^+^ → ) spectra of (A) 7β-HC, (B) 7α-HC and (C) 7-OC. Aliquots of sample prepared in the presence of cholesterol oxidase were derivatised with [^2^H_5_]GP and those prepared in the absence of cholesterol oxidase with [^2^H_0_]GP.

**Fig. 4:**
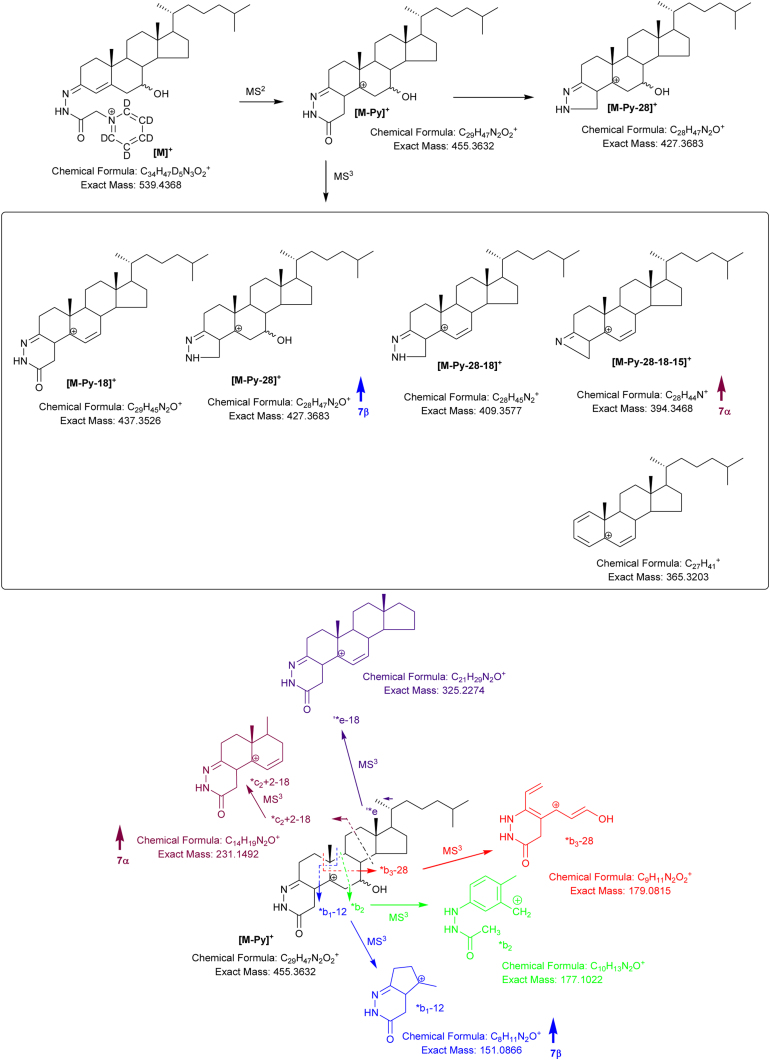
Fragmentation of 7-hydroxycholesterols. Ions of elevated relative abundance in the spectra of 7α- and 7β- epimers are indicated by arrows.

The MS^3^ spectra of 7β-HC and 7α-HC/7α-HCO are similar but not identical (Fig. 3). The 7α-hydroxy group is more labile with the result that the [M-Py-18]^+^ fragment-ion (*m*/*z* 437, Fig. 4) dominates the MS^3^ spectrum and the [M-Py-28-18-15]^+^ (*m*/*z* 394) and *c_2_ + 2-18 (*m*/*z* 231) fragment-ions are of greater relative abundant than in the MS^3^ spectrum of 7β-HC. On the other hand, the [M-Py-28]^+^ (*m*/*z* 427) and *b_1_-12 (*m*/*z* 151) fragment-ions are of higher relative abundance in the spectrum of 7β-HC than 7α-HC (Figs. 3A and B & 4).

The abundance of 7α-HC and 7β-HC are of diagnostic value. One example is cerebrotendinous xanthomatosis (CTX) where the enzyme CYP27A1 is deficient resulting in reduced levels of its product (25R)26-hydroxycholesterol ((25R)26-HC) and greatly elevated levels of 7α-HC and 7α-HCO in plasma (Fig. 2C and D) (DeBarber et al., 2011). In SLOS where both 7-DHC and 7-OC are elevated in plasma so is 7β-HC formed from 7-OC by HSD11B1 (Fig. 2E and F). A complication when using 7-HC epimers or 7-OC in disease diagnosis is that each can be formed in air by reaction with oxygen (Fig. 2G and H) (Schroepfer, 2000).

#### 3.1.2 24S-HC and 24R-HC

**Fig. 5:**
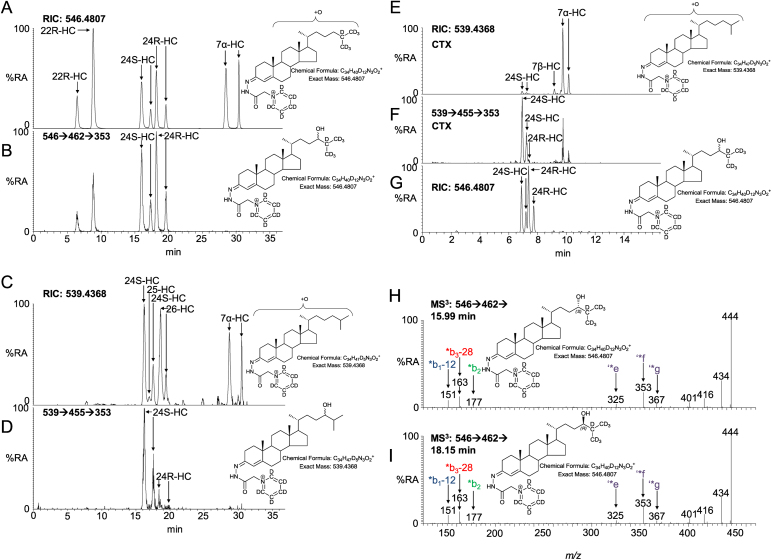
Separation and fragmentation of 24S-HC and 24R-HC. (A) RIC for 24S-HC and 24R-HC using a 37 min gradient. Other peaks correspond to 22R-HC and 7α-HC. The hydroxycholesterols (HCs) are heavy isotope labelled with [25,26,26,26,27,27,27-^2^H_7_] and derivatised with [^2^H_5_]GP. (B) MRM chromatogram for the [M]^+^ → [M-Py]^+^ → 353.3 ± 0.3 transition. 22R-HC also shows a fragment ion at *m*/*z* 353.3 (see Supplementary Fig. S1A). (C) RIC for monohydroxycholesterols in a typical human serum sample and (D) MRM chromatogram for the [M]^+^ → [M-Py]^+^ → 353.3 ± 0.3 transition, highlighting 24-HC, in the same sample. (E) RIC for HCs in a CTX plasma sample using a 17 min gradient. (F) MRM chromatogram for the [M]^+^ → [M-Py]^+^ → 353.3 ± 0.3 transition from the CTX patient using the 17 min gradient (Crick et al., 2015b). (G) RIC for [^2^H_7_]24S-HC and [^2^H_7_]24R-HC using the 17 min gradient. (H) MS^3^ ([M^+^ → [M-Py]^+^→) spectra of [^2^H_7_]24S-HC and (I) [^2^H_7_]24R-HC. Note in the spectra of the [^2^H_7_]24-HC molecules fragment ions are observed at *m*/*z* 444, 434, 416 and 401 which are 7 Da heavier than equivalent fragment ions in the [^2^H_0_]24-HC native molecules (see Supplementary Fig. S1C & S1F and Fig. 6). Analyte concentrations are given in supplementary Table S1.

**Fig. 6:**
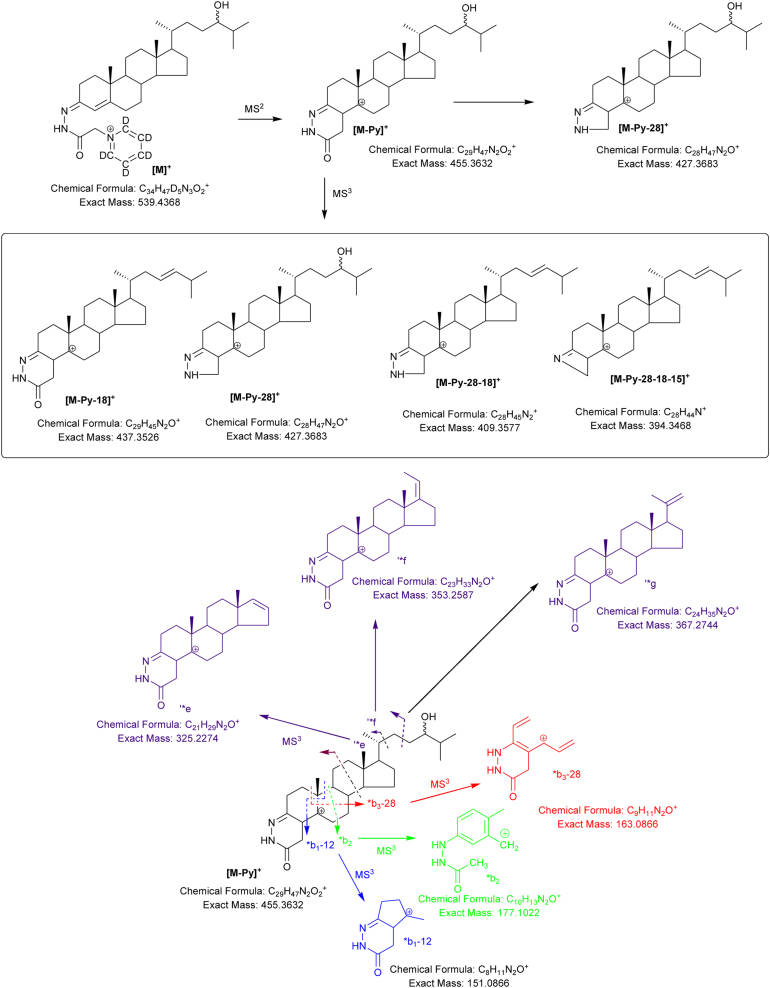
Fragmentation of 24-hydroxycholesterols.

24S-HC elutes before 24R-HC (Fig. 5A–D & E–G showing two different gradients). They give an essentially identical MS^3^ spectrum where the fragment ion ‘*f at *m*/*z* 353 is prominent (Figs. 5 H and I & 6 ). This ion is very minor in closely eluting 25-hydroxycholesterol (25-HC) and (25R)26-HC (see Supplementary Fig. S1). By performing a RIC for *m*/*z* 353 in the MS^3^ chromatogram to give a multiple reaction monitoring (MRM) chromatogram for transitions [M]^+^ → [M-Py]^+^ → 353 the 24-HC epimers are highlighted. This is demonstrated for [25,26,26,26,27,27,27-^2^H_7_]24*S*-HC and [25,26,26,26,27,27,27-^2^H_7_]24*R*-HC authentic standards in Fig. 5B (MS^3^ spectra shown in Fig. 5H and I) and in a typical human serum sample in Fig. 5D and in a CTX plasma sample in Fig. 5F. The fragmentation pattern for the endogenous molecule is described by Fig. 6 and its MS^3^ spectrum shown in supplementary Fig. S1. Quantification can be performed using the MRM chromatograms for the authentic standard and endogenous molecules.

#### 3.1.3 3,7-Dihydroxycholest-5-en-26-oic and 7-Hydroxy-3-oxocholest-4-en-26-oic acids

**Fig. 7:**
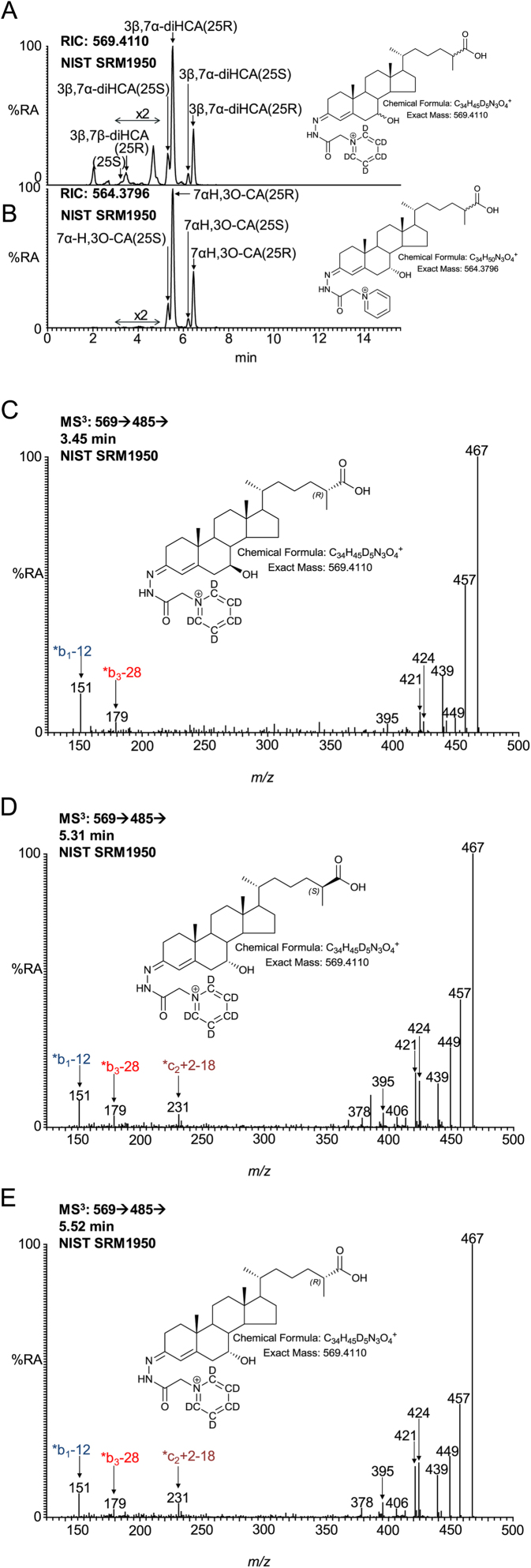
Separation and fragmentation of diastereoisomers of 3,7-dihydroxycholest-5-en-26-oic and 7-hydroxy-3-oxocholest-4-en-26-oic acids. (A) RIC from NIST SRM1950 plasma showing the separation of 3β,7β-diHCA(25S), 3β,7β-diHCA(25R), 3β,7α-diHCA(25S) and 3β,7α-diHCA(25R). Peak intensities in the time window 3–5 min have been multiplied by 2. (B) The 3β,7β-diHCA epimers are not substrates for HSD3B7 so do not give 7βH,3O-CA compounds, while 3β,7α-diHCA are and give 7αH,3O-CA epimers. (C) MS^3^ ([M]^+^ → [M-Py]^+^ → ) spectra of 3β,7β-diHCA(25R), (D) 3β,7α-diHCA(25S) and (E) 3β,7α-diHCA(25R). Aliquots of sample prepared in the presence of cholesterol oxidase were derivatised with [^2^H_5_]GP and those prepared in the absence of cholesterol oxidase with [^2^H_0_]GP. Analyte concentrations are given in supplementary Table S1.

Both 3β,7α-dihydroxycholest-5-en-(25*R*)26-oic (3β,7α-diHCA(25R)) and 7α-hydroxy-3-oxocholest-4-en-(25*R*)-26-oic (7αH,3O-CA(25R)) acids are found in plasma (Fig. 7A and B). They can be formed via both the neutral and acidic pathways of bile acid biosynthesis (Russell, 2003). Once a 7α-hydroxy group is present on the B-ring, the sterol becomes a substrate for HSD3B7 and the 3β-hydroxy-5-ene is converted to a 3-oxo-4-ene. Both acids when derivatised give double peaks corresponding to *syn* and *anti* confirmers of the GP-hydrazone. Surprisingly, in plasma a quartet rather than a doublet of peaks is observed in the appropriate RIC, each giving an identical MS^3^ spectrum (Fig. 7D and E). Custom synthesis of [^2^H_2_]-labelled 25R and 25S epimers (supplementary Fig. S2) showed that the smaller peaks of the quartet correspond to the 25S epimers, 3β,7α-diHCA(25S) and 7αH,3O-CA(25S), while the larger peaks correspond to the 25R epimers.

**Fig. 8:**
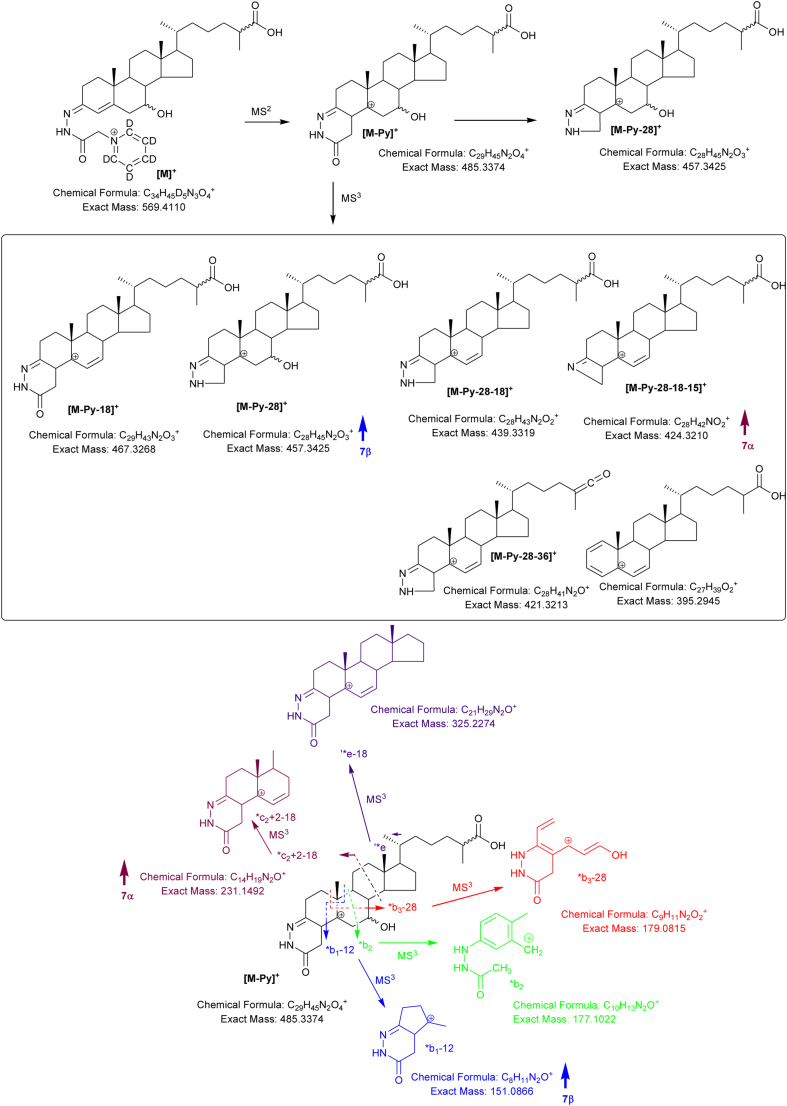
Fragmentation of 3β,7α-diHCA and 3β,7β-diHCA. Ions of elevated relative abundance in the spectra of 7α- and 7β- epimers are indicated by arrows.

Further possible diastereomers are 3β,7β-dihydroxycholest-5-en-(25*R*)26-oic (3β,7β-diHCA(25R)) and 3β,7β-dihydroxycholest-5-en-(25*S*)26-oic (3β,7β-diHCA(25S)). As HSD3B7 is not active on sterols with a 7β-hydroxy group the 7β-hydroxy-3-oxo-4-ene acids are not expected to present endogenously (Furster et al., 1996). Close scrutiny of the RIC shown in Fig. 7A reveals a peak with retention time and giving an MS^3^ spectrum (Fig. 7C) identical to the 3β,7β-diHCA(25R) authentic standard. This peak is absent in Fig. 7B where the sample was prepared in the absence of cholesterol oxidase. In keeping with the MS^3^ spectra of 7α-HC and 7-βHC, the [M-Py-18]^+^ ion at *m*/*z* 467 dominates the spectrum of the 7α-epimers, with the [M-Py-28-18-15]^+^ (*m*/*z* 424) and *c_2_ + 2-18 (*m*/*z* 231) being of greater relative abundance than in the spectrum of the 7β-epimer (Fig. 8). On the other hand the fragment ions [M-Py-28]^+^ (*m*/*z* 457) and *b_1_-12 (*m*/*z* 151) are of greater relative abundance in the spectrum of the 7β-epimer. An early eluting shoulder can be observed on the chromatographic peak assigned to the 3β,7β-diHCA(25R) diastereomer. This shoulder is absent in a chromatogram of the authentic 3β,7β-diHCA(25R) standard, but gives an identical MS^3^ spectrum. It is thus assigned to 3β,7β-diHCA(25S).

#### 3.1.4 3,7-Dihydroxychol-5-en-24-oic and 7-Hydroxy-3-oxochol-4-en-24-oic acids

**Fig. 9:**
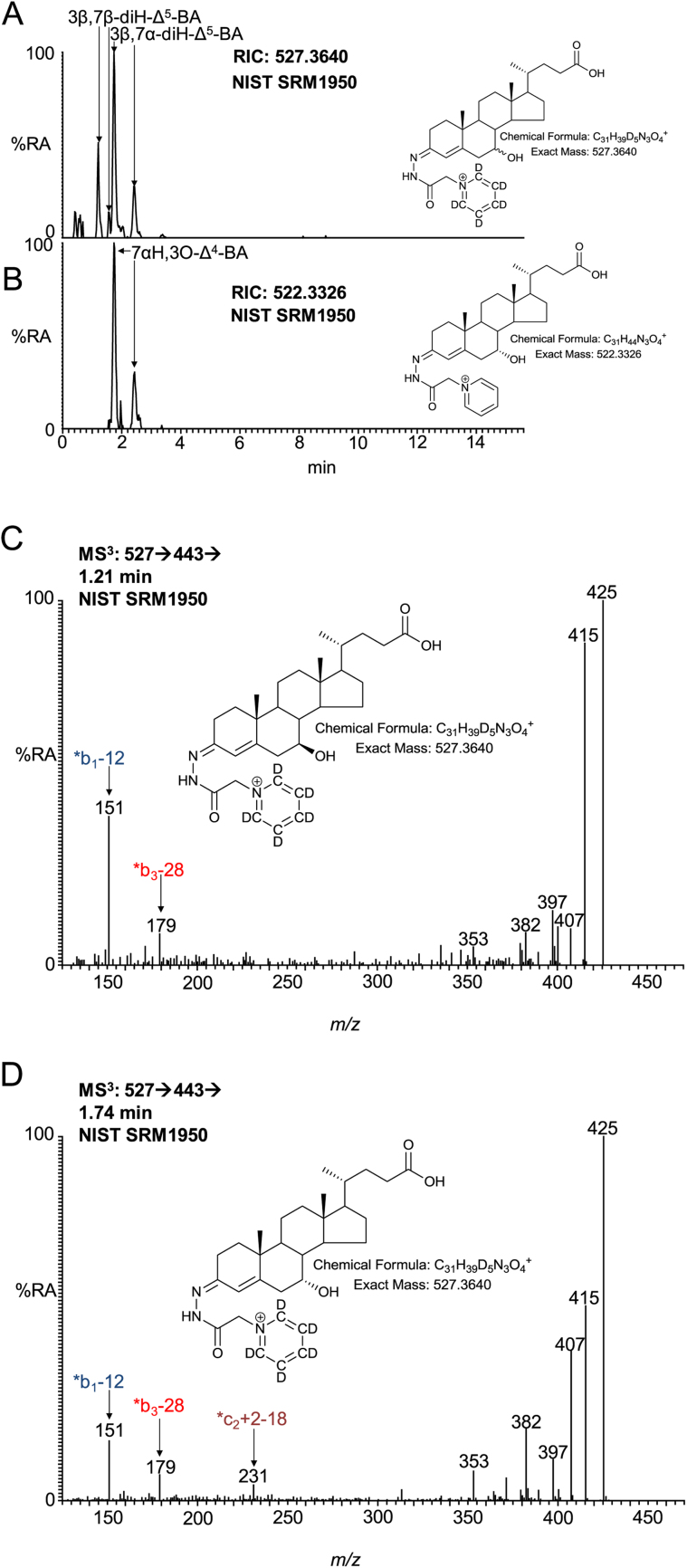
Separation and fragmentation of diastereoisomers of 3,7-dihydroxychol-5-en-24-oic and 7-hydroxy-3-oxochol-4-en-24-oic acids. (A) RIC from NIST SRM1950 plasma showing the separation of 3β,7β-diH-Δ^5^-BA and 3β,7α-diH-Δ^5^-BA. (B) The 3β,7β-diH-Δ^5^-BA is not a substrate for HSD3B7 so does not give 7βH,3O-Δ^4^-BA, while 3β,7α-diH-Δ^5^-BA is and gives 7αH,3O-Δ^4^-BA. MS^3^ ([M]^+^ → [M-Py]^+^ → ) spectra of (C) 3β,7β-diH-Δ^5^-BA and (D) 3β,7α-diH-Δ^5^-BA. Aliquots of sample prepared in the presence of cholesterol oxidase were derivatised with [^2^H_5_]GP and those prepared in the absence of cholesterol oxidase with [^2^H_0_]GP. Analyte concentrations are given in supplementary Table S1.

**Fig. 10:**
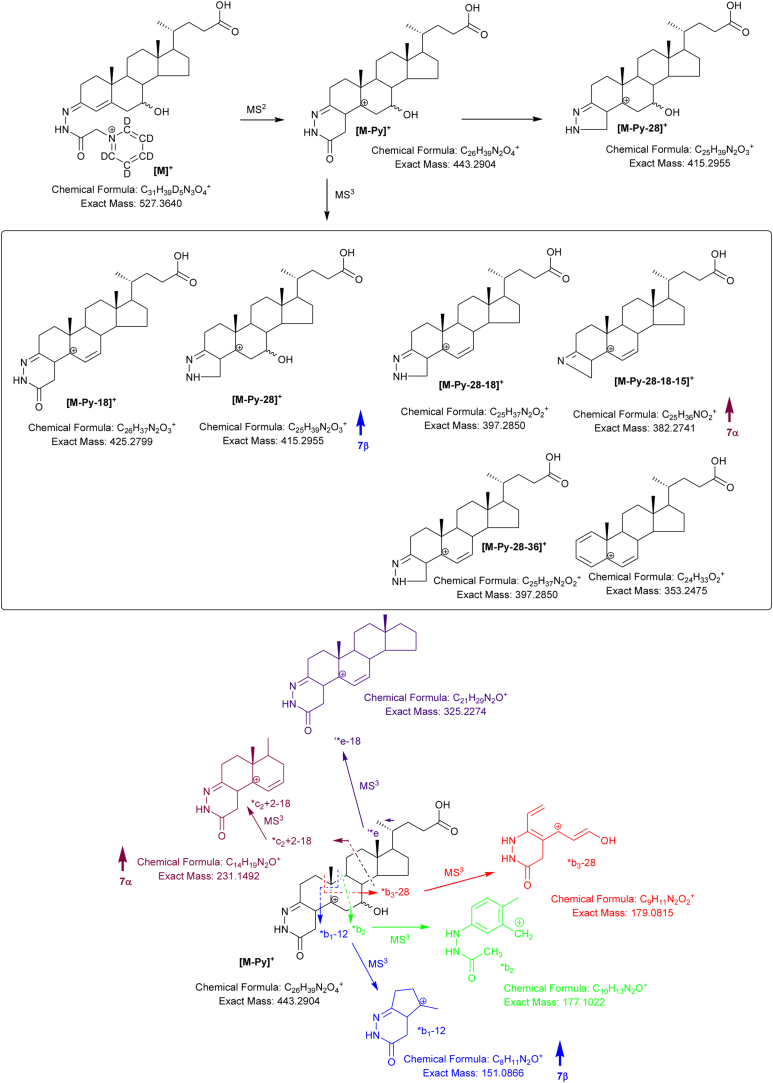
Fragmentation of 3β,7α-diH-Δ^5^-BA and 3β,7β-diH-Δ^5^-BA. Ions of elevated relative abundance in the spectra of 7α- and 7β- epimers are indicated by arrows.

After side-chain shortening in the peroxisome C_27_ 7-hydroxy acids will be converted to their C_24_ equivalents, we thus screened for the presence of 3β,7α-dihydroxychol-5-en-24-oic (3β,7α-diH-Δ^5^-BA), 7α-hydroxy-3-oxochol-4-en-24-oic (7αH,3O-Δ^4^-BA) and 3β,7β-dihydroxychol-5-en-24-oic (3β,7β-Δ^5^-BA) acids in plasma. 7β-Hydroxy-3-oxochol-4-en-24-oic acid should not be formed via HSD3B7 oxidation due to the presence of a 7β- rather than a 7α-hydroxy group (Furster et al., 1996). Shown in Fig. 9A and B is the appropriate RIC for 3β,7-diH-Δ^5^-BA and 7αH,3O-Δ^4^-BA. Comparison with authentic standards reveals that the first pair of peaks in Fig. 9A correspond to 3β,7β-diH-Δ^5^-BA (*syn* and *anti* GP conformers) while the latter correspond to 3β,7α-diH-Δ^5^-BA. As expected only the latter pair of peaks corresponding to 7αH,3O-Δ^4^-BA are observed in Fig. 9B recorded on the sample prepared in the absence of cholesterol oxidase. MS^3^ spectra of the 3β,7β-diH-Δ^5^-BA and 3β,7α-diH-Δ^5^-BA epimers are shown in Fig. 9C and D and as illustrated in Fig. 10 show distinctive features of the respective 7β- and 7α-epimers.

## 4 Discussion

It has previously been shown that 7β-HC can be formed not only ex *vivo* through autoxidation during sample handling (Schroepfer, 2000), but also in *vivo* enzymatically from 7-OC by HSD11B1 (Hult et al., 2004, Larsson et al., 2007, Mitic et al., 2013) and perhaps also by free radical reactions from cholesterol (Murphy and Johnson, 2008) (Fig. 1). In fact, 7β-HC is elevated in plasma from people with SLOS who also show elevated 7-OC (Griffiths et al., 2016) and also in patients suffering from NPC disease who similarly show elevated 7-OC in plasma (data not shown). Interestingly, Alvelius et al. showed that bile acids with a 3β,7β-diH-Δ^5^-BA core structure were formed by a patient with NPC disease (Alvelius et al., 2001), while Evans and Natowicz also found bile acids probably with the same structure formed by a patient with SLOS (Natowicz and Evans, 1994). 7β-HC may represent an early metabolite in the biosynthesis of these acids. Using our optimised EADSA approach we also find comparatively (to the 7α-epimers) low levels of 7β-HC, 3β,7β-diHCA and 3β,7β-diH-Δ^5^-BA in plasma from healthy individuals, see Supplementary Table S1 (Abdel-Khalik et al., 2017).

In healthy individuals, we identify four diastereoisomers for 3β,7-diHCA. Besides the 25R epimers of 3β,7α-diHCA and 3β,7β-diHCA we also identify the corresponding 25S epimers. The interconversion of 25R and 25S epimers as CoA thioesters is catalysed by AMACR, the next enzyme in the bile acid biosynthesis pathway ACOX2 requiring 25S stereochemistry of the substrate (Russell, 2003). It has recently been reported that a defect in ACOX2 leads to liver fibrosis, mild ataxia, and cognitive impairment (Vilarinho et al., 2016). A blockage in the bile acid biosynthesis pathway at this point would be expected to lead to elevated levels of both epimers of 3β,7α-diHCA and 3β,7β-diHCA in plasma which could easily be determined by the EADSA method.

24S-HC is the dominant oxysterol in brain. It is formed by CYP46A1 mediated oxidation of cholesterol (Russell, 2003). The origin of the 24R-epimer is less clear. Interestingly, 24R-HC is present in brain of the CYP46A1 knockout mouse indicating a source other than a reaction catalysed by CYP46A1 (Meljon et al., 2014).

## 5 Conclusions

We describe here how the EADSA method has been optimised to reveal diastereoisomers of oxysterols, cholestenoic and cholenoic acids. Our observations highlight the extreme complexity of the plasma sterolome.
