# Supplementary material for: Charge-tagging liquid chromatography–mass spectrometry methodology targeting oxysterol diastereoisomers
Source: Chem Phys Lipids. 2017 Oct;207(Pt B):69–80. doi: 10.1016/j.chemphyslip.2017.04.004 (PMC5630687; doi:10.1016/j.chemphyslip.2017.04.004)
Supplement: Supplementary file 1 [file mmc1.pdf]

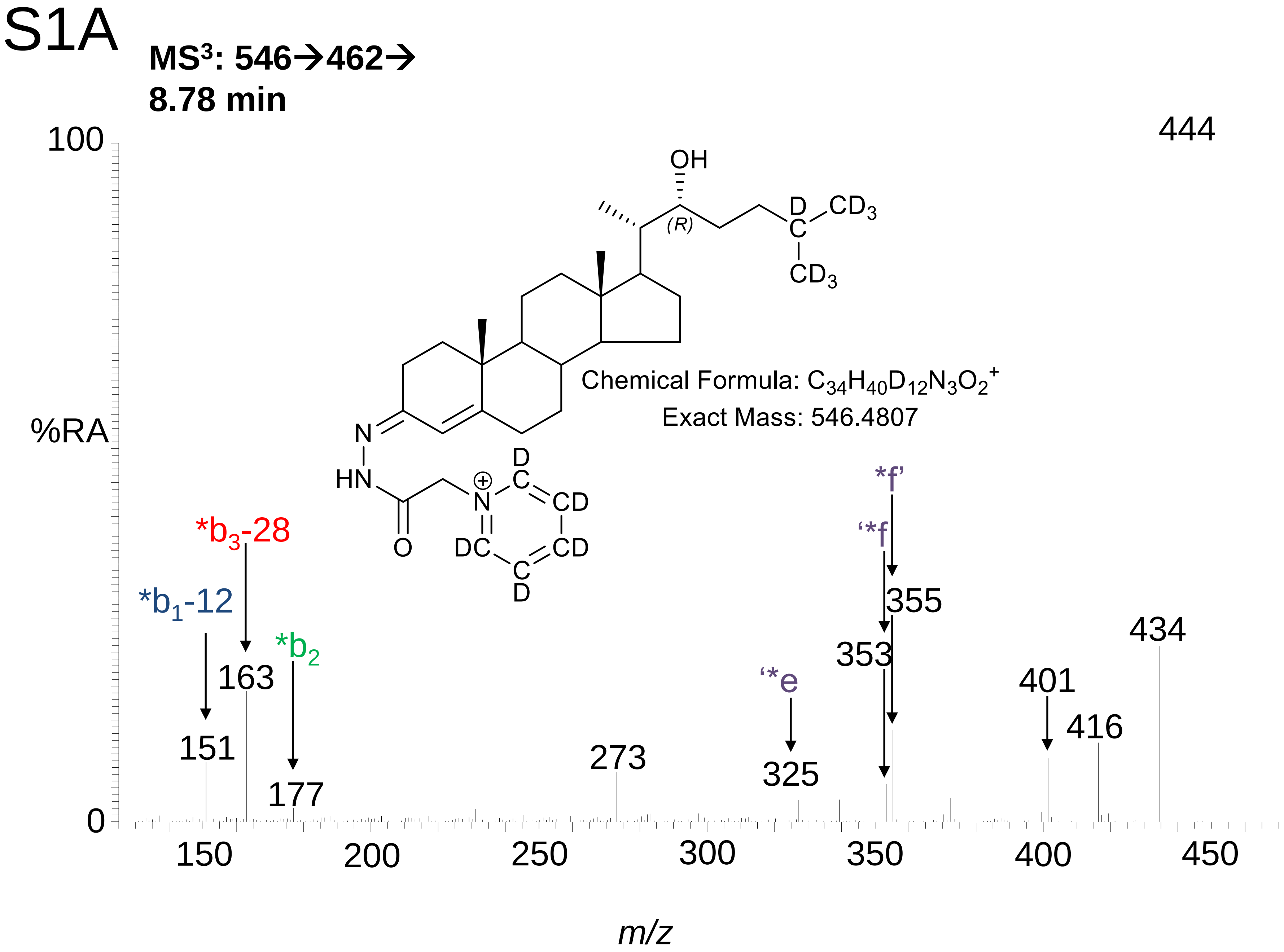
S1A
MS³: 546→462→
8.78 min
100
%RA
0
OH
(R)
D
CD3
C
CD3
N
HN
O
N
D
C
CD
CD
DC
C
D
Chemical Formula: C34H40D12N3O2+
Exact Mass: 546.4807
444
*f'
'*f
355
353
434
*b3-28
*b1-12
163
*b2
151
177
273
'*e
325
401
416
150
200
250
300
350
400
450
m/z

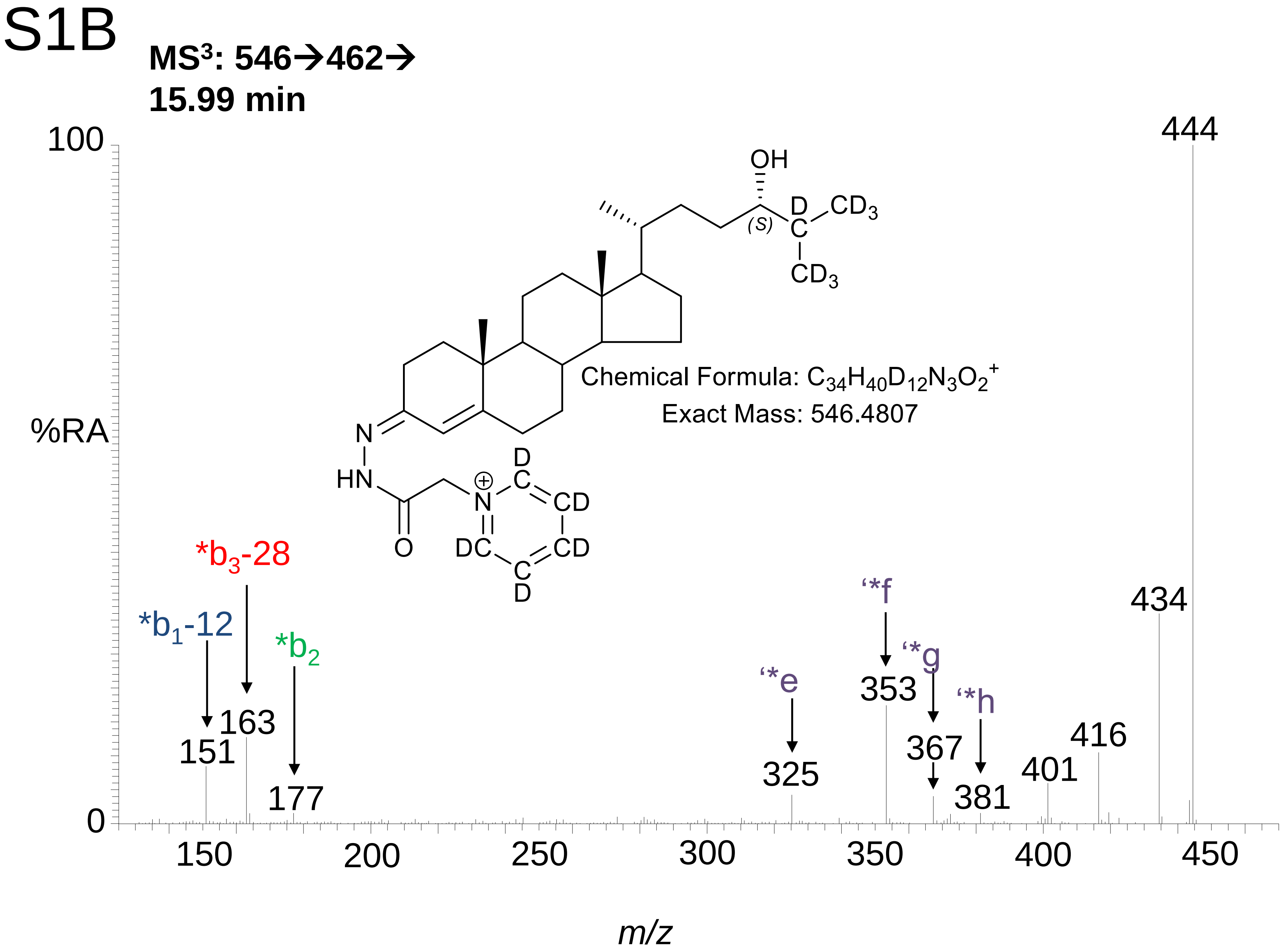
S1B
MS3: 546→462→
15.99 min
100
%RA
0
444
434
416
401
381
367
353
325
177
163
151
*b1-12
*b3-28
*b2
‘*e
‘*f
‘*g
‘*h
OH
(S)
D
C
CD3
CD3
N
HN
O
N
D
C
CD
CD
DC
C
D
Chemical Formula: C34H40D12N3O2+
Exact Mass: 546.4807
150
200
250
300
350
400
450
m/z

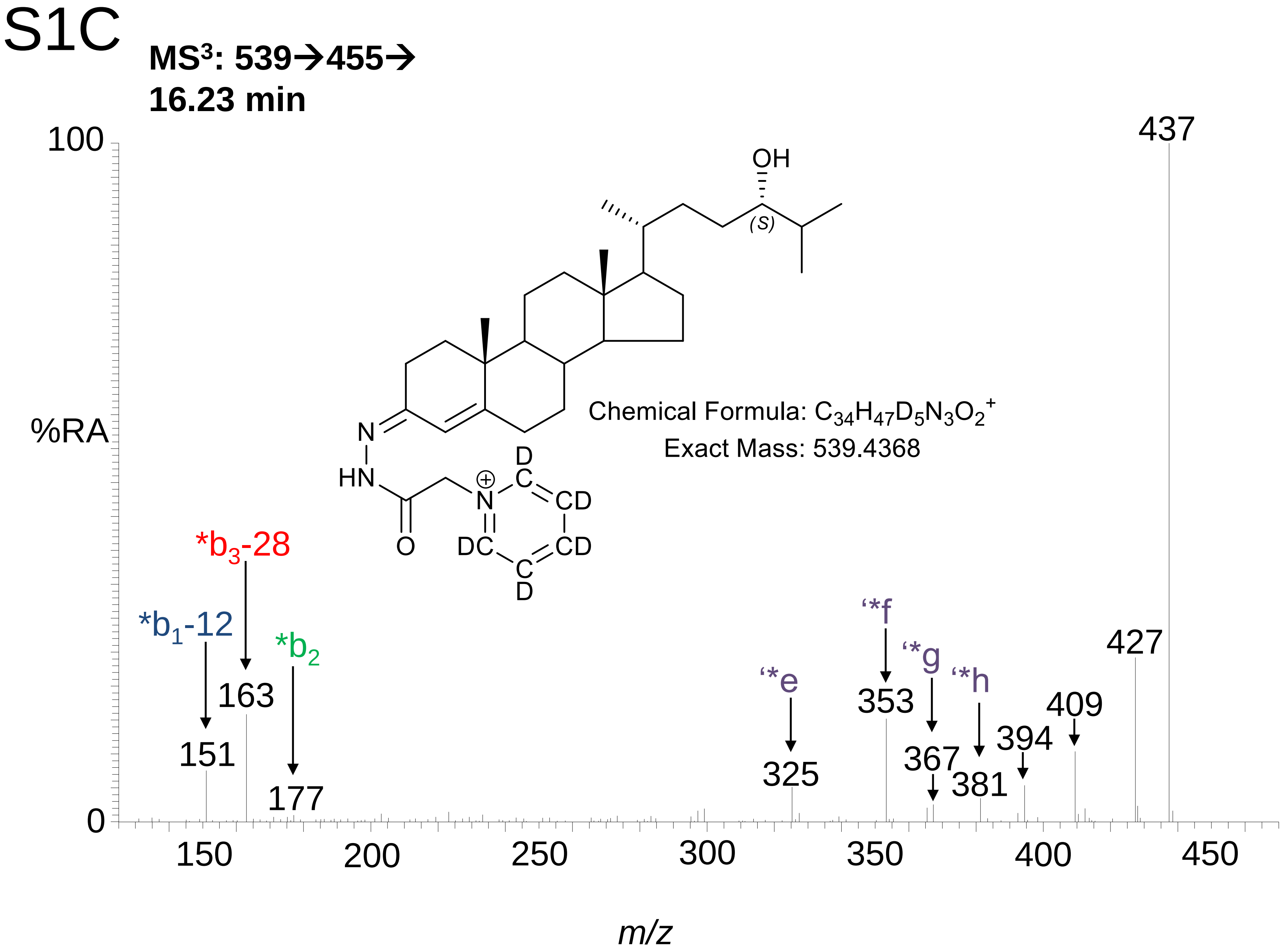

S1C
MS3: 539→455→
16.23 min
100
%RA
0
437
427
409
394
381
367
353
325
177
163
151
*b3-28
*b1-12
*b2
‘*e
‘*f
‘*g
‘*h
150
200
250
300
350
400
450
m/z
OH
(S)
N
HN
O
N
D
C
CD
CD
DC
C
D
Chemical Formula: C34H47D5N3O2+
Exact Mass: 539.4368

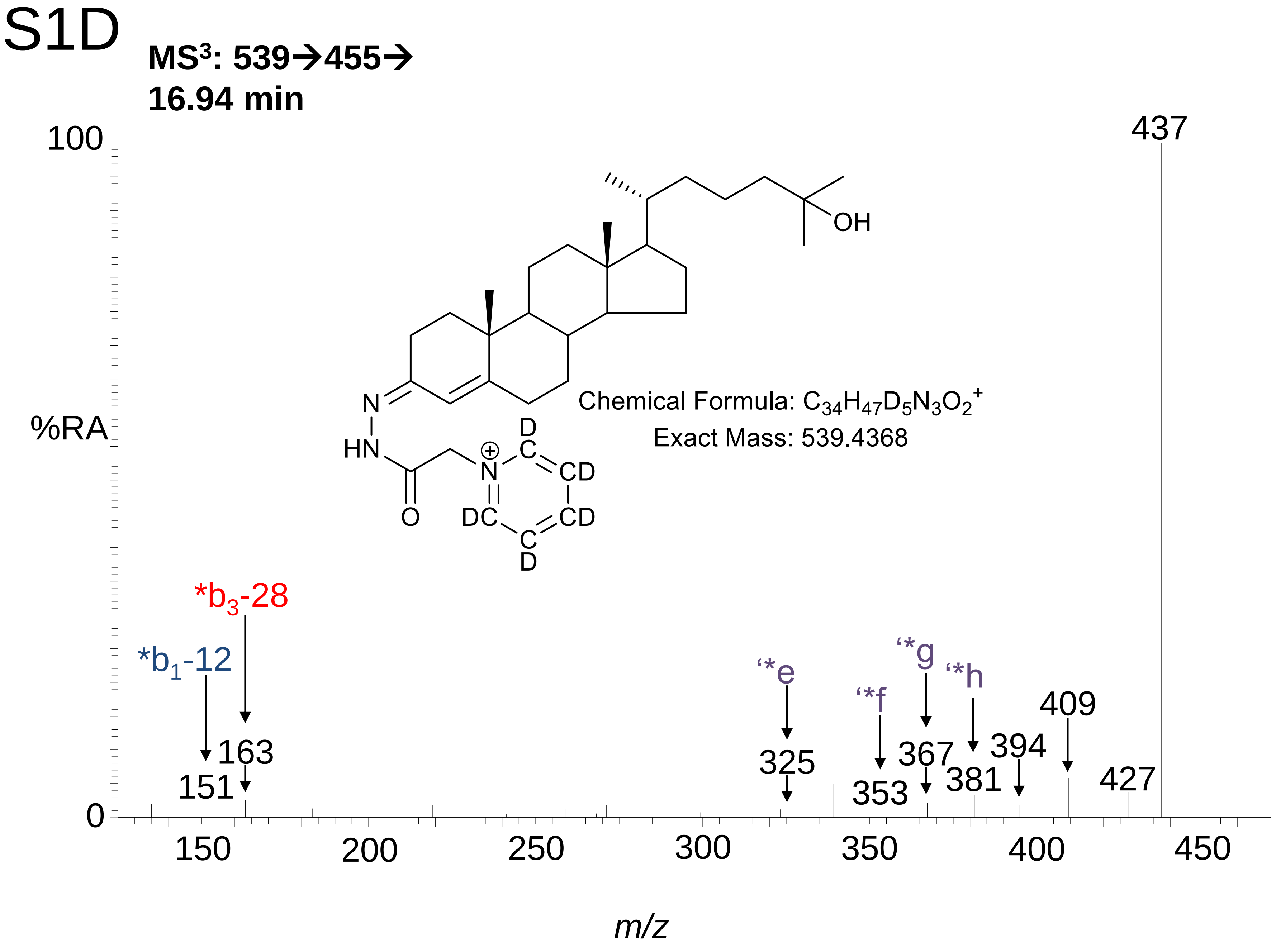
S1D
MS3: 539→455→
16.94 min
100
%RA
0
437
Chemical Formula: C34H47D5N3O2+
Exact Mass: 539.4368
OH
N
HN
O
N
D
C
CD
CD
DC
C
D
*b3-28
*b1-12
163
151
'*e
325
'*f
353
'*g
367
'*h
381
394
409
427
150
200
250
300
350
400
450
m/z

S1E

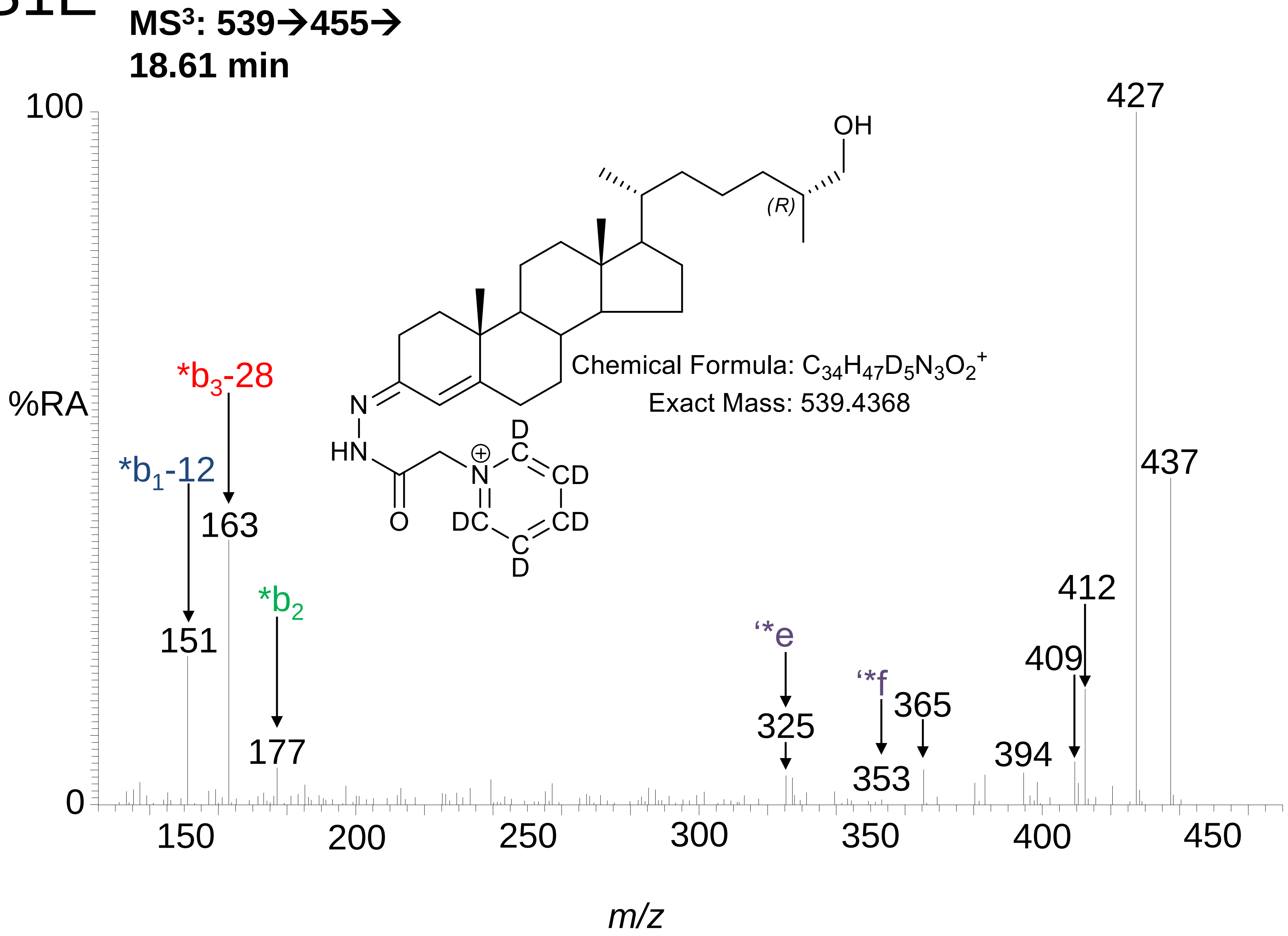

MS³: 539→455→
18.61 min
100
%RA
0
427
437
OH
(R)
Chemical Formula: C34H47D5N3O2+
Exact Mass: 539.4368
N
HN
O
N
D
C
CD
CD
DC
C
D
*b3-28
163
*b1-12
151
*b2
177
'*e
325
'*f
353
365
394
409
412
150
200
250
300
350
400
450
m/z

S1F

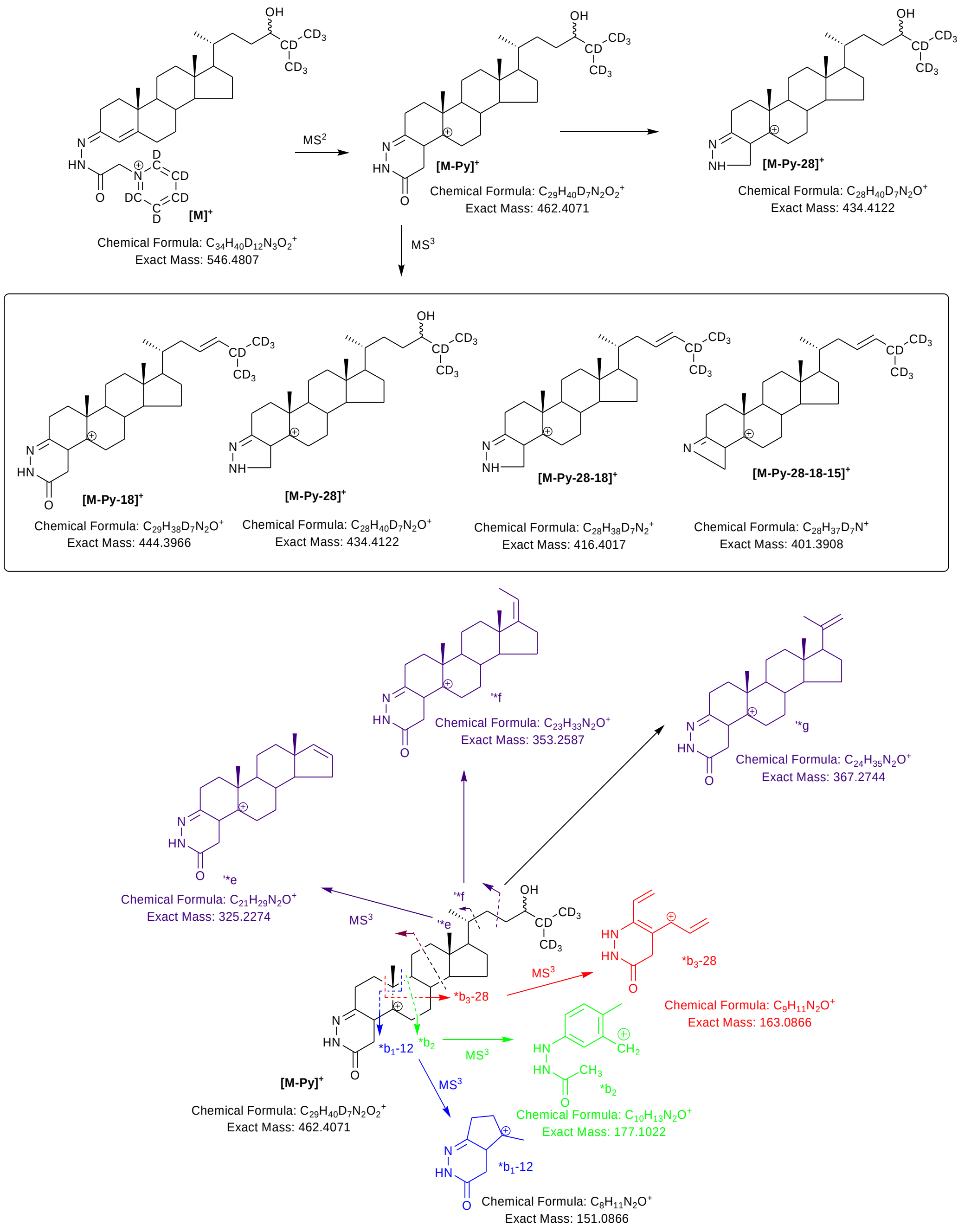

[M]+
Chemical Formula: C34H40D12N3O2+
Exact Mass: 546.4807
MS2
[M-Py]+
Chemical Formula: C29H40D7N2O2+
Exact Mass: 462.4071
[M-Py-28]+
Chemical Formula: C28H40D7N2O+
Exact Mass: 434.4122
MS3
[M-Py-18]+
Chemical Formula: C29H38D7N2O+
Exact Mass: 444.3966
[M-Py-28]+
Chemical Formula: C28H40D7N2O+
Exact Mass: 434.4122
[M-Py-28-18]+
Chemical Formula: C28H38D7N2+
Exact Mass: 416.4017
[M-Py-28-18-15]+
Chemical Formula: C28H37D7N+
Exact Mass: 401.3908
*f
Chemical Formula: C23H33N2O+
Exact Mass: 353.2587
*g
Chemical Formula: C24H35N2O+
Exact Mass: 367.2744
*e
Chemical Formula: C21H29N2O+
Exact Mass: 325.2274
MS3
*b3-28
Chemical Formula: C9H11N2O+
Exact Mass: 163.0866
*b1-12
*b2
[M-Py]+
Chemical Formula: C29H40D7N2O2+
Exact Mass: 462.4071
*b2
Chemical Formula: C10H13N2O+
Exact Mass: 177.1022
*b1-12
Chemical Formula: C8H11N2O+
Exact Mass: 151.0866

S2A

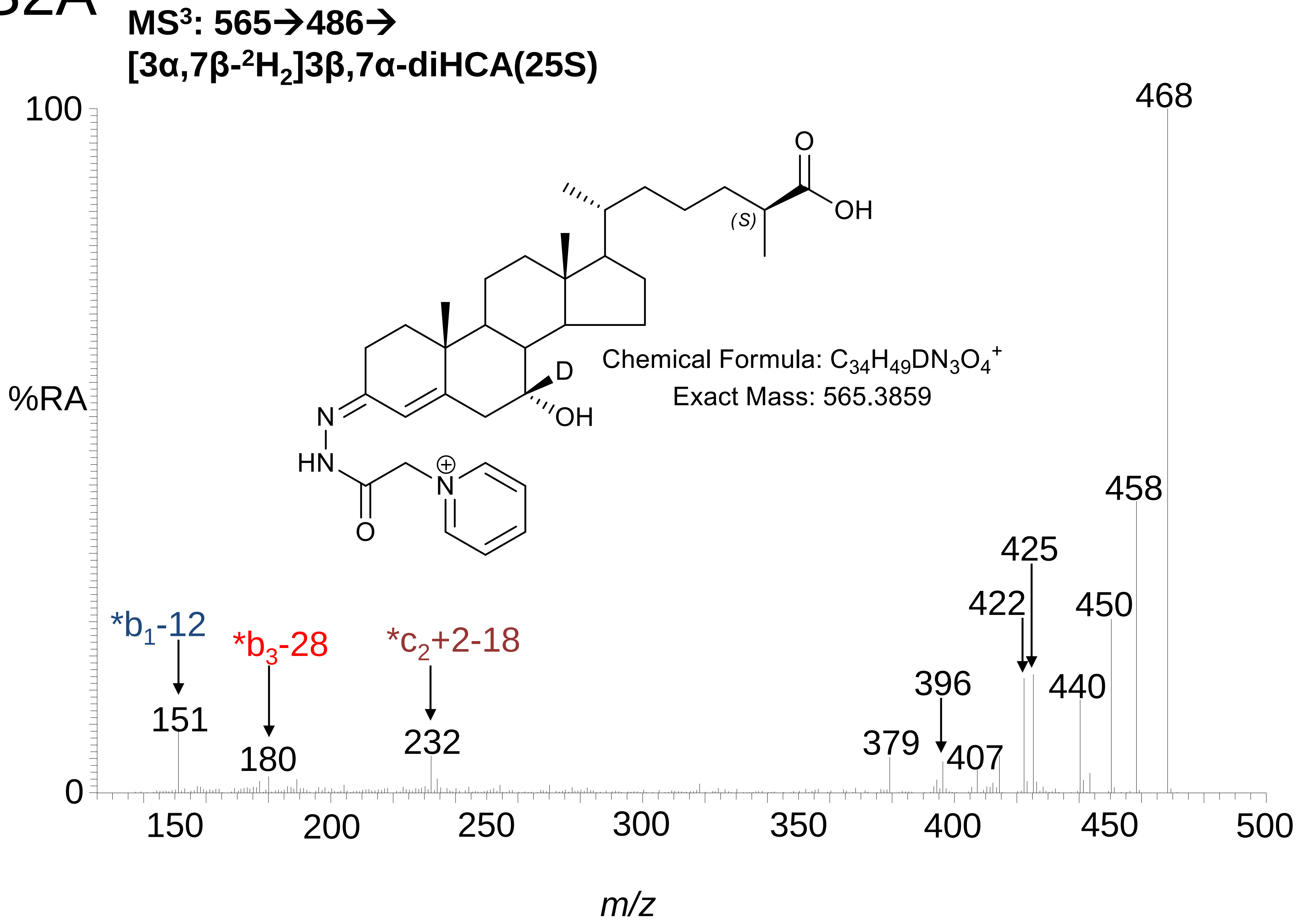
MS3: 565→486→
[3α,7β-2H2]3β,7α-diHCA(25S)
100
%RA
0
Chemical Formula: C34H49DN3O4+
Exact Mass: 565.3859
(S)
OH
O
D
OH
N
HN
N
O
*b1-12
*b3-28
*c2+2-18
151
180
232
379
396
407
422
425
440
450
458
468
150
200
250
300
350
400
450
500
m/z

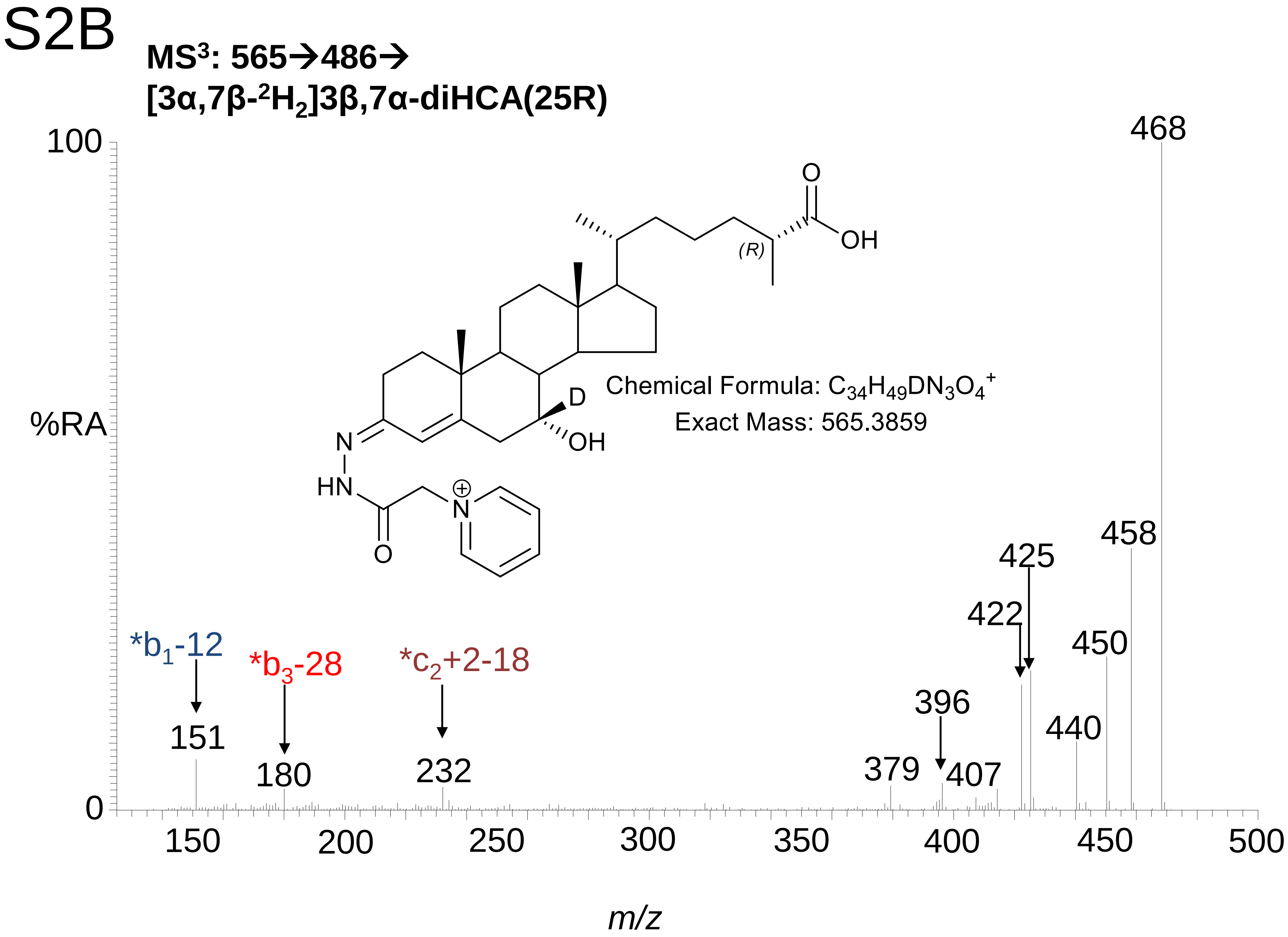

S2B
MS3: 565→486→
[3α,7β-2H2]3β,7α-diHCA(25R)
Chemical Formula: C34H49DN3O4+
Exact Mass: 565.3859
%RA
100
0
m/z
*b1-12
*b3-28
*c2+2-18
151
180
232
379
396
407
422
425
440
450
458
468
150
200
250
300
350
400
450
500
O
OH
(R)
D
OH
N
HN
N
O

S2C

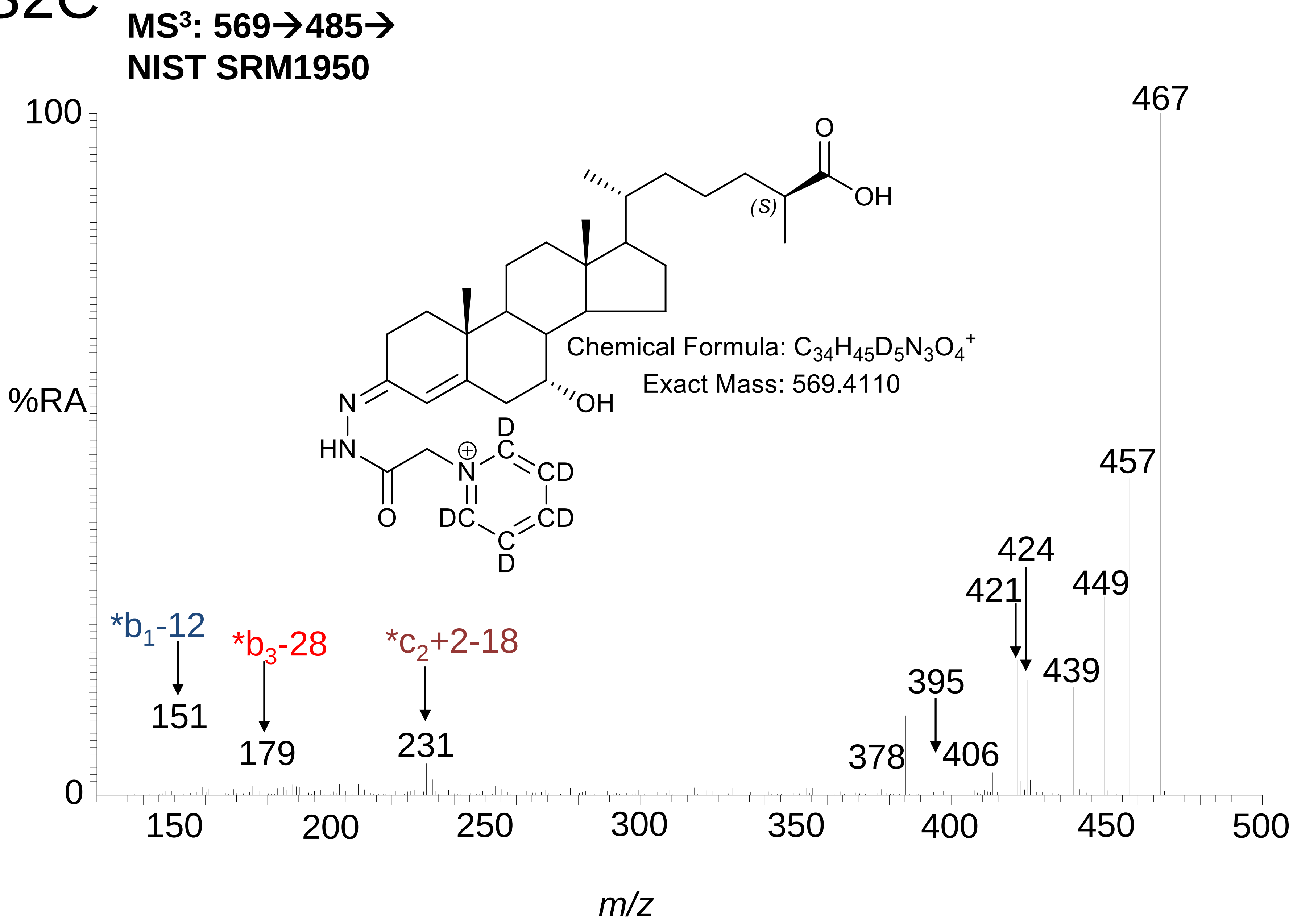
MS³: 569→485→
NIST SRM1950
100
%RA
0
467
457
449
424
421
439
395
406
378
*b₁-12
151
*b₃-28
179
*c₂+2-18
231
Chemical Formula: C34H45D5N3O4+
Exact Mass: 569.4110
O
OH
(S)
OH
N
HN
O
N
D
C
CD
CD
DC
C
D
150
200
250
300
350
400
450
500
m/z

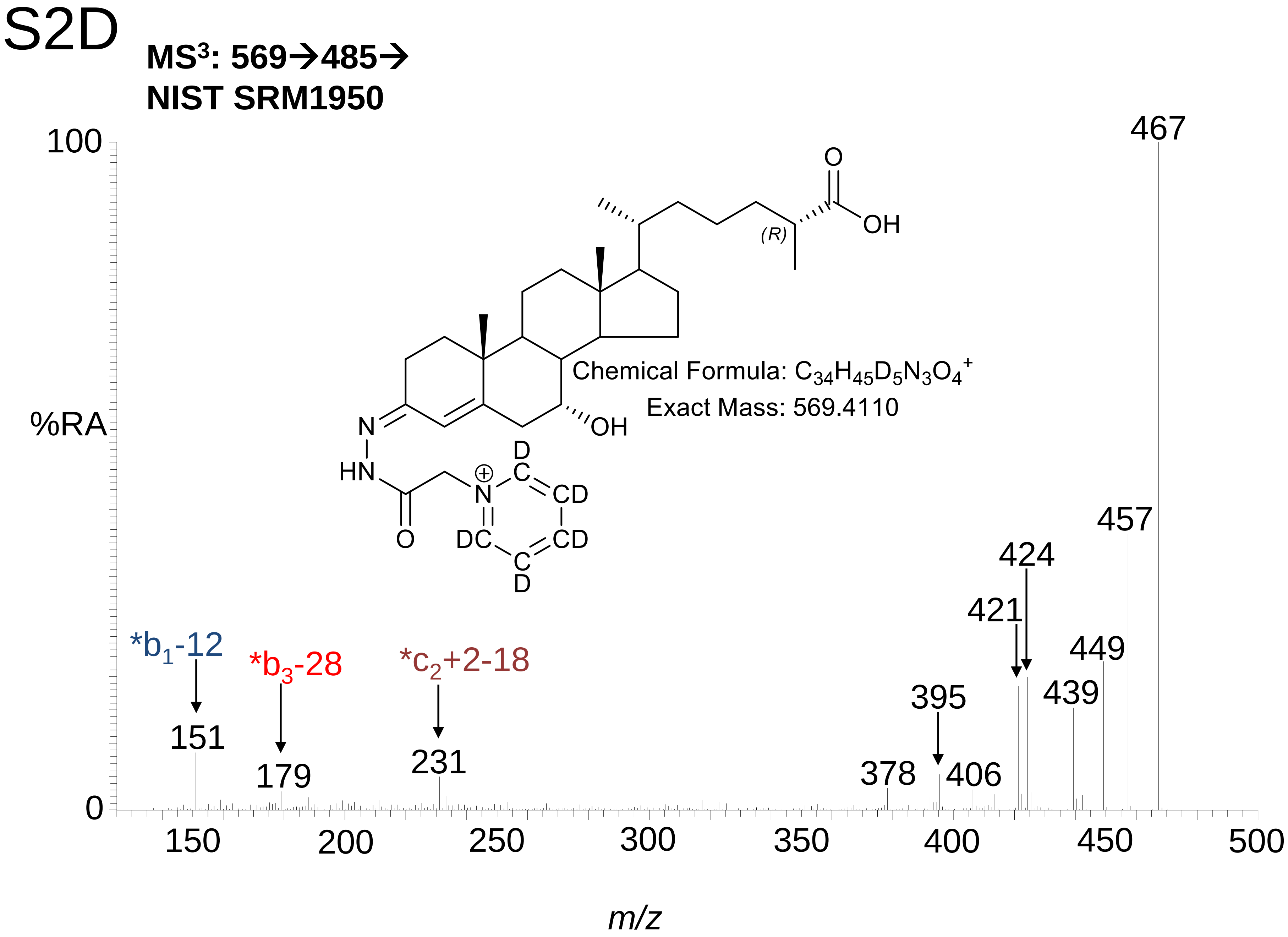
S2D
MS³: 569→485→
NIST SRM1950
100
%RA
0
150
200
250
300
350
400
450
500
m/z
467
457
424
421
449
439
395
378
406
*b₁-12
151
*b₃-28
179
*c₂+2-18
231
(R)
O
OH
N
HN
O
D
C
CD
DC
C
D
Chemical Formula: C₃₄H₄₅D₅N₃O₄⁺
Exact Mass: 569.4110

S2F

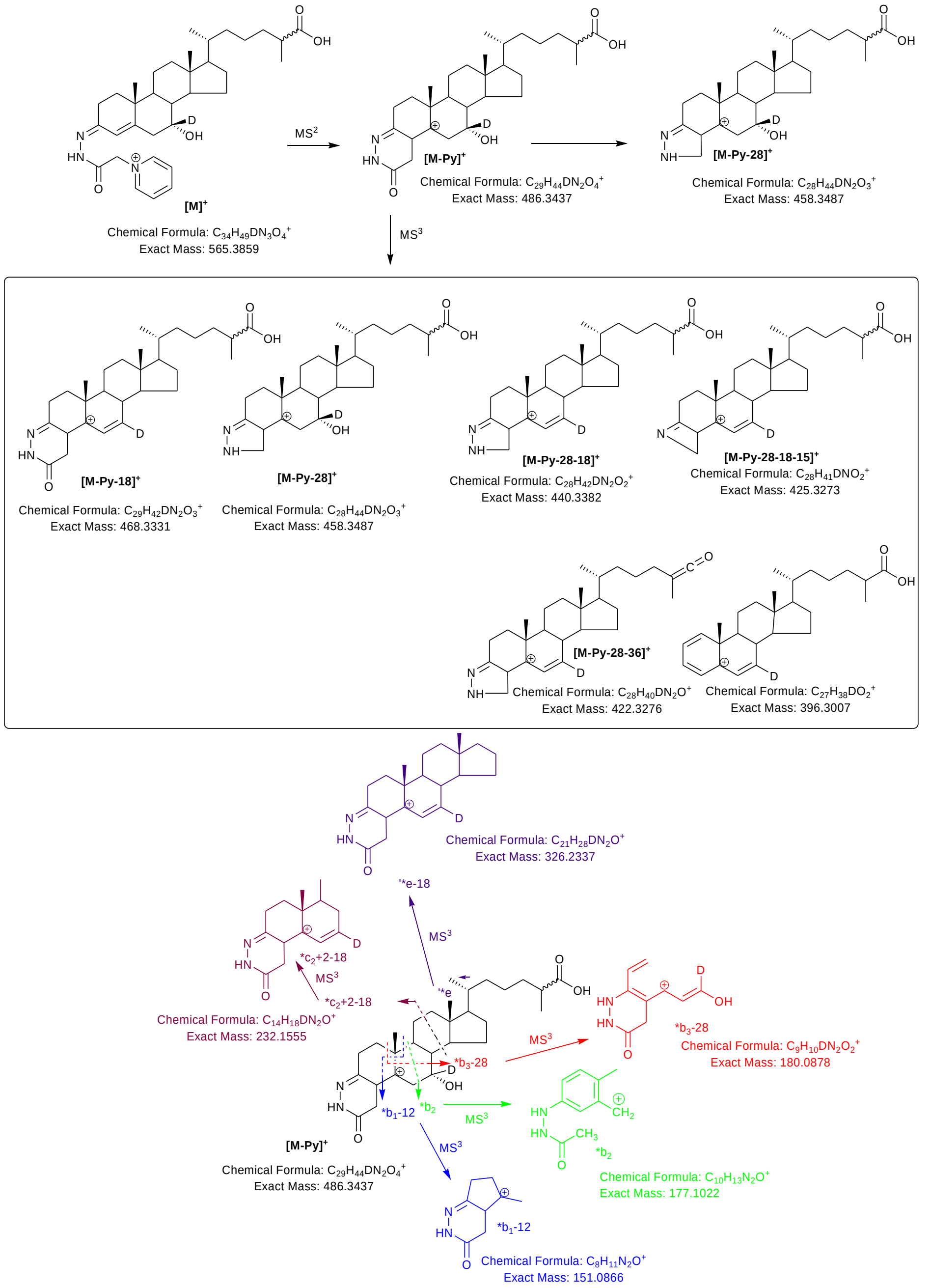

[M]+
Chemical Formula: C34H49DN3O4+
Exact Mass: 565.3859
MS2
[M-Py]+
Chemical Formula: C29H44DN2O4+
Exact Mass: 486.3437
[M-Py-28]+
Chemical Formula: C28H44DN2O3+
Exact Mass: 458.3487
MS3
[M-Py-18]+
Chemical Formula: C29H42DN2O3+
Exact Mass: 468.3331
[M-Py-28]+
Chemical Formula: C28H44DN2O3+
Exact Mass: 458.3487
[M-Py-28-18]+
Chemical Formula: C28H42DN2O2+
Exact Mass: 440.3382
[M-Py-28-18-15]+
Chemical Formula: C28H41DNO2+
Exact Mass: 425.3273
[M-Py-28-36]+
Chemical Formula: C28H40DN2O+
Exact Mass: 422.3276
Chemical Formula: C27H38DO2+
Exact Mass: 396.3007
Chemical Formula: C21H28DN2O+
Exact Mass: 326.2337
*e-18
*c2+2-18
Chemical Formula: C14H18DN2O+
Exact Mass: 232.1555
*b3-28
Chemical Formula: C9H10DN2O2+
Exact Mass: 180.0878
*b2
Chemical Formula: C10H13N2O+
Exact Mass: 177.1022
*b1-12
Chemical Formula: C8H11N2O+
Exact Mass: 151.0866
[M-Py]+
Chemical Formula: C29H44DN2O4+
Exact Mass: 486.3437
