# Supplementary material for: Charge-tagging liquid chromatography–mass spectrometry methodology targeting oxysterol diastereoisomers
Source: Chem Phys Lipids. 2017 Oct;207(Pt B):69–80. doi: 10.1016/j.chemphyslip.2017.04.004 (PMC5630687; doi:10.1016/j.chemphyslip.2017.04.004)
Supplement: Supplementary file 2 [file mmc2.pdf]

Supplementary Table S1. Concentrations of some, oxysterols, cholestenoic and cholenoic acids in plasma or serum

Sterol Systematic Name (Common name)	Abbreviation	Control serum, n=24		Reference	NIST SRM1950 plasma, n=3		Reference	SLOS plasma, n=10		Reference	CTX plasma, n=4		Reference
		Mean	SD		Mean	SD		Mean	SD		Mean	SD	
**Metabolites**		ng/mL			ng/mL			ng/mL			ng/mL		Theofilopoulos et al., 2014
Cholest-5-ene-3β,7α-diol (7α-Hydroxycholesterol)	7α-HC	5.91	5.25	Abdel-Khalik et al., 2017	9.52	2.64	Griffiths et al., 2013a	5.30	5.54	Griffiths et al., 2016	78.25	102.12	Theofilopoulos et al., 2014
7α-Hydroxycholest-4-en-3-one	7α-HCO	22.60	21.11	Abdel-Khalik et al., 2017	10.01	1.65	Griffiths et al., 2013a	1.29	1.39	Griffiths et al., 2016	70.77	79.17	Theofilopoulos et al., 2014
Cholest-5-ene-3β,7β-diol (7β-Hydroxycholesterol)	7β-HC	1.12	0.63	Abdel-Khalik et al., 2017	0.48	0.28	Griffiths et al., 2013a	12.33	12.12	Griffiths et al., 2016	24.01	15.04	Theofilopoulos et al., 2014
3β-Hydroxycholest-5-en-7-one (7-Oxocholesterol)	7O-C	2.70	1.95	Abdel-Khalik et al., 2017	0.59	0.33	Griffiths et al., 2013a	41.29	51.44	Griffiths et al., 2016	34.35	48.30	Theofilopoulos et al., 2014
Cholest-5-ene-3β,24S-diol (24S-Hydroxycholesterol)	24S-HC	8.23	2.28	Abdel-Khalik et al., 2017	5.46	0.05	Griffiths et al., 2013a	8.70	7.43	Griffiths et al., 2016	10.11	7.53	Theofilopoulos et al., 2014
Cholest-5-ene-3β,24S-diol (24S-Hydroxycholesterol)	24R-HC	NA			0.62		Present work	NA			NA		
Cholest-5-ene-3β,25-diol (25-Hydroxycholesterol)	25-HC	1.31	0.47	Abdel-Khalik et al., 2017	0.70	0.02	Griffiths et al., 2013a	1.23	1.28	Griffiths et al., 2016	3.60	2.46	Theofilopoulos et al., 2014
Cholest-5-ene-3β,(25R)26-diol (27-Hydroxycholesterol)	26-HC	23.17	4.83	Abdel-Khalik et al., 2017	10.33	0.60	Griffiths et al., 2013a	10.99	7.04	Griffiths et al., 2016	0.00		Theofilopoulos et al., 2014
Total 3β,7α-Dihydroxycholest-5-en-(25R)26-oic acid	3β,7α-diHCA(25R)	23.69	7.94	Abdel-Khalik et al., 2017	19.22	1.92	Griffiths et al., 2013a	NA			0.00		Theofilopoulos et al., 2014
Total 7α-Hydroxy-3-oxocholest-4-en-(2SR)26-oic acid	7αH,3O-CA(25R)	66.88	15.93	Abdel-Khalik et al., 2017	58.27	5.48	Griffiths et al., 2013a	NA			0.00		Theofilopoulos et al., 2014
Total 3β,7α-Dihydroxycholest-5-en-(25S)26-oic acid	3β,7α-diHCA(25S)	NA			6.53		Present work	NA			NA		
Total 7α-Hydroxy-3-oxocholest-4-en-(25R)26-oic acid	7αH,3O-CA(25S)	NA			7.72		Present work	NA			NA		
3β,7β-Dihydroxycholest-5-en-26-oic acid	3β,7β-diHCA(25R)	5.30	0.89	Abdel-Khalik et al., 2017	2.74	0.18	Griffiths et al., 2013a	NA			0.00		Theofilopoulos et al., 2014
3β,7β-Dihydroxycholest-5-en-26-oic acid	3β,7β-diHCA(25S)	NA			0.41		Present work	NA			NA		Theofilopoulos et al., 2014
3β,7α-Dihydroxychol-5-en-24-oic acid	3β,7α-Δ^5-BA	2.10	1.04	Abdel-Khalik et al., 2017	1.04	0.10	Griffiths et al., 2013a	NA			0.00		
7α-Hydroxy-3-oxochol-4-en-24-oic acid	7αH,3O-Δ^4-BA	3.05	1.02	Abdel-Khalik et al., 2017	2.03	0.27	Griffiths et al., 2013a	NA			0.00		
3β,7β-Dihydroxychol-5-en-24-oic acid	3β,7β-Δ^5-BA	1.26	0.44	Abdel-Khalik et al., 2017	0.97		Present work	NA			NA		Theofilopoulos et al., 2014

Notes

1. SD, standard deviation
2. NA, not available
